# Methodological Aspects of Indirect Calorimetry in Patients with Sepsis—Possibilities and Limitations

**DOI:** 10.3390/nu14050930

**Published:** 2022-02-22

**Authors:** Weronika Wasyluk, Agnieszka Zwolak, Joop Jonckheer, Elisabeth De Waele, Wojciech Dąbrowski

**Affiliations:** 1 Department of Internal Medicine in Nursing, Faculty of Health Sciences, Medical University of Lublin, Witold Chodźko Street 7, 20-093 Lublin, Poland; agnieszka.zwolak@umlub.pl; 2 Doctoral School, Medical University of Lublin, 20-093 Lublin, Poland; 3 Intensive Care, UZ Brussel, University Hospital Brussels (UZB), 1090 Jette, Belgium; joop.jonckheer@uzbrussel.be (J.J.); elisabeth.dewaele@uzbrussel.be (E.D.W.); 4 Department of Nutrition, UZ Brussel, University Hospital Brussels (UZB), 1090 Jette, Belgium; 5 Faculty of Medicine and Pharmacy, Vrije Universiteit Brussel, 1090 Brussels, Belgium; 6 Department of Anaesthesiology and Intensive Care, Medical University of Lublin, 20-954 Lublin, Poland; wojciech.dabrowski@umlub.pl

**Keywords:** sepsis, septic shock, energy expenditure, energy demand, calorimetry, metabolism, clinical nutrition, critical care

## Abstract

The aim of the review was to analyse the challenges of using indirect calorimetry in patients with sepsis, including the limitations of this method. A systematic review of the literature was carried out. The analysis concerned the methodology and presentation of research results. In most studies assessing energy expenditure, energy expenditure was expressed in kcal per day (*n* = 9) and as the mean and standard deviation (*n* = 7). Most authors provided a detailed measurement protocol, including measurement duration (*n* = 10) and device calibration information (*n* = 7). Ten papers provided information on the day of hospitalisation when the measurements were obtained, nine on patient nutrition, and twelve on the criteria for inclusion and exclusion of participants from the study. Small study group sizes and study at a single centre were among the most cited limitations. Studies assessing energy expenditure in patients with sepsis by indirect calorimetry differ in the methodology and presentation of results, and their collective analysis is difficult. A meta-analysis of the results could enable multi-site and large patient evaluation. Standardisation of protocols and presentation of all collected data would enable their meta-analysis, which would help to achieve greater knowledge about metabolism in sepsis.

## 1. Introduction

Sepsis is defined as a “life-threatening organ dysfunction caused by a dysregulated host response to infection” [1]. Dysregulated host response includes, among others, changes in basic metabolic processes and limitations in the body’s metabolic capacity [2,3]. In addition, hormonal disorders and circulating cytokines occurring in sepsis cause insulin resistance and promote lipolysis and proteolysis, the latter of which can lead to cachexia [3,4]. This endogenous energy production is not measureable in clinical practice and therefore only 70% of resting energy expenditure (REE) should be given during the first days. The metabolic changes induced by sepsis itself and the initiated therapies [5] are only measurable by indirect calorimetry as the predicting equations do not take these into consideration. Therefore, according to both the European Society for Clinical Nutrition and Metabolism (ESPEN) and the American Society for Parenteral and Enteral Nutrition (ASPEN), indirect calorimetry (IC) is the recommended method for assessing the energy needs of critically ill, mechanically ventilated patients [6,7,8,9,10].

However several issues compromise the use of IC during sepsis: oxygen enrichment (FiO_2_ > 60%), organ support therapies such as renal replacement or liver support therapy and extracorporeal membrane oxygenation (ECMO) (may change blood gas levels or affect acid-base balance), unstable pH, and unstable body temperature [10].

The aim of the study was to analyse these challenges of using IC in patients with sepsis and to highlight limitations of this method that may reduce its clinical utility and limit reliability of studies using it. The presentation of the methodology and results of this analysis will be preceded by a summary of current knowledge about the evolution of the definition of sepsis, and the theoretical basis of IC. This information is essential for a thorough understanding of the limitations described below.

## 2. Materials and Methods

A review of the literature was carried out between March and May 2020. The literature search process was conducted as for a systematic review in accordance with the PRISMA guidelines [11]. Articles were searched for in PubMed/Medline database. The following search formula was used: (sepsis [Title/Abstract]) OR (septic [Title/Abstract]) AND (indirect calorimetry [Title/Abstract]). The review was carried out in several stages. In the first stage, the titles and abstracts of the papers were reviewed to exclude review articles, congress annals, guidelines, case reports, studies not using IC and studies that did not include patients with sepsis/septic shock, or in which the inclusion criteria remained unclear. In the case of any doubt, the entire article was analysed before making a decision. In the second stage, the full content of articles describing research on sepsis and using IC was read, after which animal studies, studies with children, and studies with healthy subjects were excluded. After reviewing the content of the articles, studies older than 20 years were also excluded from the detailed analysis due to insufficient data being up to date.

The inclusion criteria for studies included in the final analysis were original articles describing studies using the IC method in adult (with the exception of two studies in which the patient age range was 15–85 years) patients diagnosed with sepsis or septic shock, with full texts in English and not older than 20 years. Exclusion criteria were no use of IC in the study, no participation of patients with sepsis/septic shock in the study, unclear criteria for inclusion in the study, animal studies, studies with children, studies with healthy subjects, and studies older than 20 years. 

A data extraction form has been designed and used for extract data from eligible studies.After completing each form, data compliance with the study was checked. The data from the forms has been compiled in tabular form, which has been partially presented in this review. The synthesis of results was carried out in a descriptive form, comparing individual aspects of the analysed studies. The discussion summarises the main results of the review, their potential practical application, and the advantages and limitations of this review.

## 3. Theory

### 3.1. Energy Expenditure

Energy expenditure (EE) has three main components: basal energy expenditure (BEE), diet-induced thermogenesis (DIT), and activity-induced energy expenditure (AEE). BEE is the amount of energy required to maintain basic metabolic activity of cells and life functions, such as respiration and body temperature; DIT is the heat production associated with substrate oxidation during energy uptake (EN and PN); while AEE is the EE associated with physical activity. These three components can be combined to form successive levels of EE–the sum of BEE and DIT is resting energy expenditure (REE), and the sum of BEE, DIT, and AEE is total energy expenditure (TEE) [10,12,13]. When discussing this issue, it is worth noting that sometimes REE literature is identified as BEE because these terms are often used interchangeably [14]. However, in the strict sense, BEE means the lowest level of EE and its measurement should be performed in very demanding conditions, including post 8-h sleep, post 10-h fast (or 12-h, depending on the source of information), complete resting posture, free from physiological and mental stress, and a thermally neutral, quiet and shaded room [10,13]. The above requirements make this measurement impossible for critically ill people. By contrast, measurements of REE are accompanied by fewer constraints (to be discussed in the following section).

### 3.2. Indirect Calorimetry

**Figure 1:**
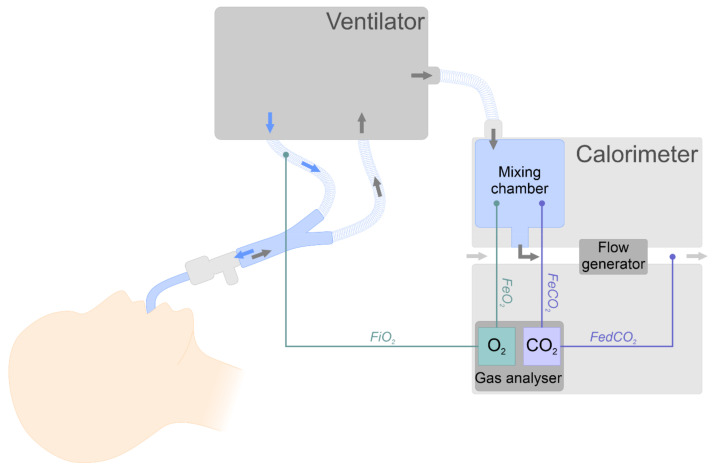
Indirect calorimetry scheme with the use of the mixing chamber technique in a mechanically ventilated patient (based on the Deltatrac Metabolic Monitor^®^ calorimeter). FiO_2_ is measured from the ventilator’s inspiratory limb, while FeO_2_ and FeCO_2_ are measured from the mixing chamber. The gas from the mixing chamber is removed through a system with a constant flow of gas, in which it is diluted with ambient air. The CO_2_ fraction in the diluted exhaust gas (FedCO_2_) is measured. VCO_2_ is calculated as the product of constant flow (Q) and FedCO_2_ (VCO_2_ = FedCO_2_ × Q). VO_2_ is calculated using the Haldane transformation. EE is calculated using the Weir equation [10,17]. Abbreviations: CO_2_—carbon dioxide; EE—energy expenditure; FeCO_2_—fraction of exhaled CO_2_; FedCO_2_—fraction of exhaled CO_2_ after dilution; FeO_2_—fraction of exhaled oxygen; FiO_2_—fraction of inhaled oxygen; O_2_—oxygen; Q—flow; VO_2_—volume of consumed oxygen; VCO_2_—volume of produced CO_2_.

IC is recognised as the gold standard for EE assessment in various populations, including in critically ill patients [6,7], cancer patients [15], and polymorbid internal medicine patients [16]. Determination of EE by the IC method requires measurement of oxygen concentration in inhaled (FiO_2_) and expired (FeO_2_) air, CO_2_ concentration in exhaled air (FeCO_2_), and the volume of exhaled gas per minute (Figure 1). These data allow the calculation of oxygen consumption (VO_2_) and CO_2_ production (VCO_2_), which can be used to calculate the EE using the Weir equation as described below [10,12].

The basis for calculations in IC is a modified Weir equation enabling the calculation of EE based on oxygen consumption (VO_2_), carbon dioxide production (VCO_2_), and urinary nitrogen (uN_2_) [18]. Due to the small share of urinary nitrogen in the actual EE in critically ill patients (the error caused by the use of respiratory functions alone was estimated at about 4%) and problematic collection of urine samples with potential additional error, a shortened version of the equation is commonly used, which omits this parameter [12,19,20]:

$$EE\;\left[\frac{kcal}{d}\right]=\left[\left(VO_{2}\times 3.941\right)+\left(VCO_{2}\times 1.11\right)\right]\times 1440$$

where: *VO*_2_—oxygen consumption [L/min]; *VCO*_2_—carbon dioxide production [L/min].

Under ideal conditions, *VO*_2_ and *VCO*_2_ could be calculated as the difference in the volume of inhaled and exhaled air multiplied by the concentrations of the respective gases as shown below:

$$VO_{2}=Vi\times FiO_{2}-Ve\times FeO_{2}\;$$

$$VCO_{2}=Ve\times FeCO_{2}-Vi\times FiCO_{2}\;$$

where: *Vi*—volume of inhaled air, *Ve*—volume of exhaled air, *FiO*_2_/*FiCO*_2_—fraction of inspired oxygen/carbon dioxide, and *FeO*_2_/*FeCO*_2_—fraction of expired oxygen/carbon dioxide.

However, in practice, due to technical difficulties in measuring the small difference between the volumes of inhaled and exhaled air, the volume of inhaled air (relatively more difficult to measure) is calculated using the Haldane transformation [12].

The Haldane transformation method is based on the assumption that nitrogen (N_2_) is not consumed or produced during breathing, so every minute the volume of inhaled N_2_ is equal to the volume of expired N_2_ [21]. Using the above assumptions, the following equations can be formulated:

$$Vi\times \left(1-FiO_{2}-FiCO_{2}\right)=Ve\times \left(1-FeO_{2}-FeCO_{2}\right)$$

It can be transformed (if *FiCO*_2_ of 0.03–0.05% is ignored) into the following equation for calculating *VO*_2_:

$$VO_{2}=\frac{\left[\left(1-FeO_{2}-FeCO_{2}\right)\times \left(FiO_{2}-FeO_{2}\right)\times Ve\right]}{\left(1-FiO_{2}\right)}$$

Haldane transformation allows the simplification of measurement systems by omitting the measurement of the volume of inhaled air. However, its use also introduces one of the main limitations in the use of IC in critically ill patients. “1−*FiO*_2_” in the denominator of the equation means that an increase in *FiO*_2_ above 60% is associated with a significant error in *VO*_2_ calculations. Therefore, in patients requiring high oxygen levels in inhaled air, the possibility of measuring EE by the IC method is limited [12,22].

### 3.3. Rules for Measuring

Correct measurement is of paramount importance for the reliability of EE measured by the IC method. The effectiveness of IC requires the following conditions to be met: proper patient preparation, correct calorimeter preparation, optimal measurement conditions, and analysis of results by experienced specialists.

Due to the demanding requirements related to patient preparation for BEE measurement (described earlier), REE measurement is usually performed for critically ill patients. This measurement should take place a minimum of five hours from a meal (or during continuous feeding), a minimum of four hours from caffeine intake, two hours from alcohol or nicotine intake, after 30 min of rest, and a minimum of two hours from moderate physical activity [10,23]. During the examination, the patient should be in a supine position and in neutral ambient conditions (temperature 27–29 °C) [10,24,25]. Some authors indicate that the measurement should take place in conditions of silence and soft lighting; however, the impact of noise on EE has not been studied so far [26,27]. In clinical situations where the possibilities of adequate preparation for the study may be limited, it is possible to liberalise, e.g., fasting time, obtaining a result sufficiently accurate for clinical purposes; however, measurements obtained in such conditions should not be used for research on REE, as they may differ as much as 100 kcal from actual REE values [23].

Technical aspects that may affect the accuracy of the results obtained include appropriate calibration and validation of the calorimeter. Accurate gas and flow rate measurements are crucial for obtaining a reliable EE. Calibration of gas analysers according to the manufacturer’s recommendations is one of the most important aspects for a correctly carried out measurement [12,27,28].

The measurement should be obtained for 30 min, or until steady state is reached. Steady state is determined by the degree of variation of VO_2_ and VCO_2_ over a period of time and can be determined when the calculated coefficient of variation (CV) % for VO_2_ and VCO_2_ is <5% within 5 min or <10% within 25 min (or 30 min, depending on the source) of the measurement. Achieving steady state is important for the accuracy of the obtained EE measurement [10,27,29,30].

When performing an EE measurement using the IC method, circumstances that could lead to inaccurate results should also be excluded. These include air leakage–patients with chest drainage, mechanically ventilated patients with high positive end expiratory pressure (PEEP > 10–12 cm H_2_O), endotracheal tube cuff leak or the presence of bronchopleural fistula may be at risk of this [10,12,31,32]. Inaccurate results can also be caused by mechanical ventilation with FiO_2_ >60%. As described earlier, due to the widespread use of a mathematical operation called the Haldane transformation, such a high concentration of oxygen in the inspiratory gas mixture can generate inaccurate results [10,12,17,22,33]. Moreover, the presence of gases other than oxygen, CO_2,_ and N_2_ in the breathing mixture (e.g., gases used in therapy or anaesthesia) may reduce the accuracy of the measurement obtained [10,12,34,35].

The usefulness of IC is also limited in patients in unstable condition because the measurement will only reflect the temporary and not the general metabolic state. Examples include agitated or involuntary patient movements, patients with unstable body temperature (>±1 °C at <1 h) or patients with unstable acid-base status. This also includes patients with changes in FiO_2_ administration, nutrient administration, or medication administration (especially in sedation and analgesia), and those who have recently undergone invasive procedures [10,12,23,35]. Additionally, some therapies used on patients may change blood gas levels or affect acid-base balance. These include extracorporeal membrane oxygenation (ECMO), renal replacement therapy, and Molecular Adsorbent Recirculating System (MARS, liver support therapy) [10,35]. The measurement of EE in patients with ECMO is theoretically possible by combining ventilator gas and ECMO analysis [36,37]. The co-authors of this review also proposed blood gas analysis from the continuous renal replacement therapy (CRRT) system as a solution enabling indirect calorimetry to be performed in patients treated with this method [38,39]. At the same time, Jonckheer et al. [39] showed that CRRT leads to a relatively small change in the measurement of EE by IC.

Interpretation and validation of test results is another important element of a properly performed EE measurement. RQ can be used as a tool to identify measurement inaccuracies. RQ < 0.7 or >1.0 (or <0.67; >1.3, depending on the source) may indicate incorrect measurement compared to the presence of air leaks, hypo- or hyperventilation, inaccuracy of the measurement system, or improper preparation of the patient for examination (prolonged fasting, excessive energy consumption, extreme pain, or agitation) [10,14,23,35,40].

The clinical context of the assessment of a metabolic state by IC should be incorporated in the interpretation of the test results, as with any medical monitor in ICU. For example, high levels of intravenous lipids in an analgesia strategy, such as propofol, should also be kept in mind when the medical nutrition prescription is designed: a large quantity of unintentional calories could be present, especially when a low concentration form of propofol is used [41].

Practical feasibility of IC in a non-research setting has been questioned for many years but has been tackled by the literature [42]. Several clinical, real life conditions can influence the results of IC. The ICALIC research group [10] discusses these influences but concludes that IC, even when influenced by small deviations due to treatment interventions, remains a better way to assess energy expenditure than predictive equations or body weight based calculations. Widespread increase in the use of this technology proves this concept.

Oshima et al. [10] proposed checkpoints for successful IC in a list that can be easily adapted to prepare a protocol for measuring EE by IC.

## 4. Results

### 4.1. Systematic Review

**Figure 2:**
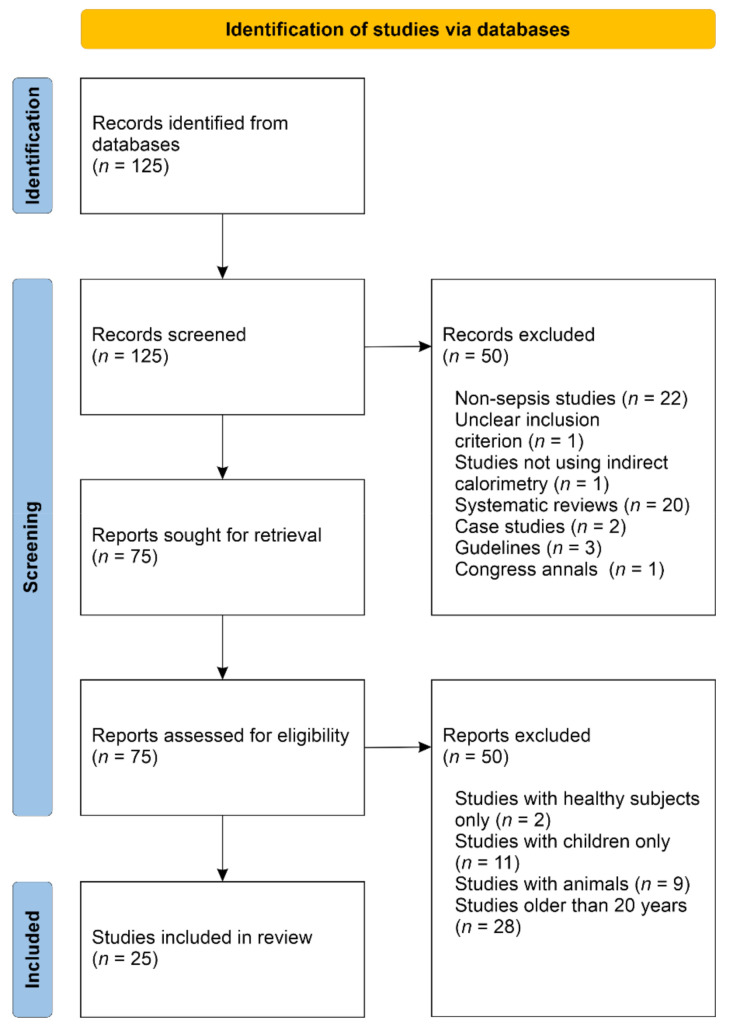
PRISMA flow diagram of the literature search process [11].

In the search conducted in PubMed/Medline, using the described methodology, 125 articles were identified. Twenty-five articles were included in this review. Both included and excluded articles are presented in the PRISMA workflow (Figure 2) [11]. The 25 articles selected represent studies in which IC was used in adult patients with sepsis or septic shock. Of these, 18 studies included patients with only sepsis or septic shock, while the remaining 7 studies included other patients, but some of them presented the data in a way that allowed analysis of data on septic patients separately.

### 4.2. Aims and Types of Research

Table 1 presents a summary of the objectives of the analysed studies. Despite the use of the IC method, only some studies aimed to assess patient EE, while in others, IC was used as a tool for measuring *VO*_2_ for the needs of the study (*n* = 9) [43,44,45,46,47,48,49,50,51]. The purpose of some studies that assessed EE was to evaluate energy metabolism in patients with sepsis (and possibly its relationship with disease severity and prognosis), compare the results obtained by various methods or develop new predictive equations. Nine such studies were identified [52,53,54,55,56,57,58,59,60]. In other studies, EE was measured to assess its change under the influence of the interventions tested–hyperinsulinaemic clamp [61,62,63], liberation from mechanical ventilation [64], continuous renal replacement therapy [65], early exercise [66], or cardiac selective beta adrenergic [67]. At least sixteen of the studies analysed were prospective. Details are presented in Table 1.

### 4.3. Energy Expenditure and Respiratory Quotient

**Figure 3:**
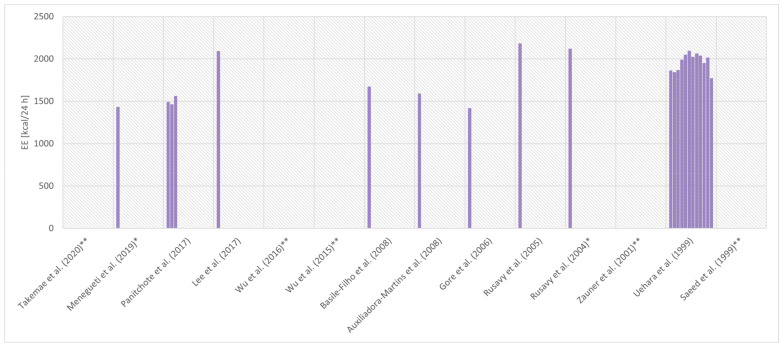
Comparison of mean energy expenditure (EE) measured by indirect calorimetry in patients with sepsis or septic shock in selected studies. [52,53,54,55,56,57,59,60,61,62,63,64,65,67] Some studies have reported more than one EE value. Time and conditions for obtaining measurements in individual tests may be different. Two studies reported the median instead of the mean. The details of the studies are presented in Table 2. * The value in the chart is the median EE. ** The authors did not publish EE at all or as kcal/24 h.

In studies assessing EE (*n* = 16), in 12 of the studies, the estimated EE was described as REE, while in the remaining four, it remained undefined. Five of the 16 EE assessments did not include patients only with sepsis, while the results of two of the studies were presented in a way that made it impossible to analyse the results of septic patients separately. The remaining 14 studies are summarised in Table 2 [68,69,70,71], which compares selected features of the examined groups (or groups of patients with sepsis in the case of studies by Menegueti et al. [53], Zauner et al. [59], and Uehara et al. [60]), sources of criteria on the basis of which sepsis/septic shock was diagnosed, type of device used in the study, day at the ICU, where the measurement was obtained, nutritional support during the measurement period, EE, and RQ results. Further analysis will involve the 14 studies presented in Table 2. In most of the studies shown in Table 2, EE was expressed in kcal per day (*n* = 9) as the mean and standard deviation (*n* = 7). The average values obtained in individual studies demonstrated a range from 1414 ± 134 kcal/day in the Gore and Wolfe study [67], to 2179 ± 354 kcal/day in the study by Rusavy et al. [61] (Figure 3).

**Figure 4:**
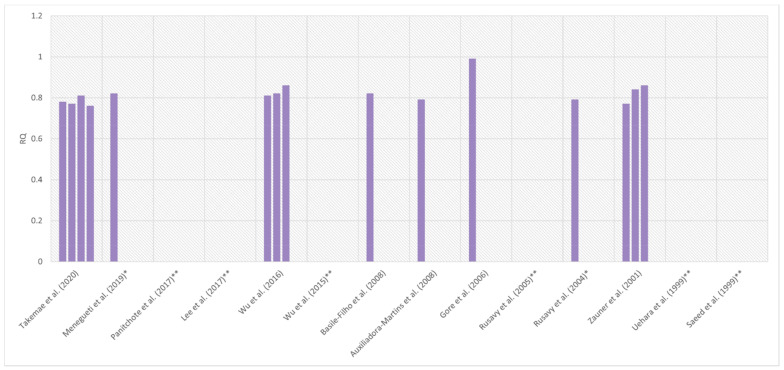
Comparison of mean respiratory quotient (RQ) measured by indirect calorimetry in patients with sepsis or septic shock in selected studies. [52,53,54,55,56,57,59,60,61,62,63,64,65,67] Some studies have reported more than one RQ value. Time and conditions for obtaining measurements in individual tests may be different. Two studies reported the median instead of the mean. The details of the studies are presented in Table 2. * The value in the chart is the median RQ. ** The authors did not publish RQ.

Some authors gave EE in kcal/kg/24 h (*n* = 2), kJ/min/ m^2^ (*n* = 1), kcal/kg FFM/min (*n* = 1), or did not provide the value at all (*n* = 2); moreover, in some papers, the results were given in the form of median and range, or median and interquartile range (*n* = 2). Of the 14 studies compared in Table 2, RQ was given in eight, of which six presented the data in the form of mean and standard deviation. The mean RQ presented in the studies ranged from 0.76 ± 0.12 [52] to 0.99 ± 0.06 [67] (Figure 4).

### 4.4. Energy Expenditure Measurement Protocol

Most study authors provided a detailed measurement protocol, including measurement duration (*n* = 10) and device calibration information (*n* = 7). Ten papers provided information on the day of hospitalisation in which the measurements were obtained (Table 2), from the measurement carried out after admission to the ward [59,65], to the twenty-third day in a multi-day study by Uehara et al. [60]. In most studies, measurements were obtained during the first week of the patient’s stay in the ward (*n* = 10). Nine articles provide information on patient nutrition during the measurement period (Table 2). In six cases, feeding was suspended prior to measurement. The number of hours for which nutrition was suspended varied depending on the study, from “at least 1.5 h” in the studies of Wu et al. [55,65], up to 9 h in the study of Rusavy et al. [62], and all-night fasting in the research of Saeed et al. [63]. In two studies, IC measurements were performed before feeding [53,59], and in one, feeding was given during the measurements [67]. All of the 14 analysed studies provided the name of the calorimetry device, which in five cases was the Deltatrac II^®^; for many years, it has been recognised as a reference device (Table 2).

### 4.5. Criteria for the Diagnosis of Sepsis and Septic Shock

Nine studies provided the source of the criteria used to diagnose sepsis and septic shock. In older studies (published up to, and including 2008), the criteria established at the ACCP and SCCM conference in 1991 (SEPSIS-1) were used. Later studies used the criteria established at the 2001 SEPSIS-2 conference and Surviving Sepsis Campaign 2008 and 2012.

### 4.6. Criteria for Participating in the Study

Of the 14 analysed articles, 12 presented information on the criteria for inclusion and exclusion of participants from the study. The most common exclusion criterion was the FiO_2_ criterion (*n* = 9), which is known to be required for a correct IC measurement result. The other exclusion criteria directly related to IC technical requirements were PEEP (*n* = 4), no chest drain (*n* = 3), and no bronchopleural fistula (*n* = 1). The criterion of the lower age limit of participants (18 (*n* = 5) or 15 (*n* = 2) years) appeared relatively often, while the criterion of the upper age limit appeared only twice (80 and 85 years). A list of exclusion criteria from individual studies is presented in Table 3.

### 4.7. Limitations of the Analysed Studies

Limitations of the described studies were given in only half (*n* = 7) of the fourteen analysed works. They are listed in Table 4. The most frequently occurring limitations included the small size of the study group (*n* = 3) (the groups in the study had 16, 27, and 62 patients) [54,55,65], as well as equal mention that the study was conducted at a single centre [53,55,65]. Doubts were also expressed as to whether one measurement per day, most often obtained at a fixed time for all patients, would be representative of daily EE [52,54,55]. In two works, it was noted that patients admitted to ICU may be at various stages of the disease, which could have initially progressed e.g. when the patient was in another ward and their condition was not severe enough to qualify them for treatment in ICU [55,64]. Some of the other limitations mentioned included: no protocol to control patient nutrition [52], EE measurement only on admission [53], difficulties in obtaining steady state [54], variability in sedation management and body mass [64], and no assessment of the impact of medical procedures on EE [55].

## 5. Discussion

### 5.1. Indirect Calorimetry

Considering the importance of accurate determination of EE in patients with sepsis, a review of the literature was carried out, trying to compare the methodology of previous studies using IC in this group of patients. A total of 25 papers meeting accepted criteria were identified, but only 14 of them described EE in patients with sepsis or septic shock. Others used IC as the method to determine VO_2_ for the needs of the study, or referred to a broader group of patients, and results for patients with sepsis could not be distinguished.

### 5.2. Energy Expenditure

Fourteen studies measuring EE in patients with sepsis were analysed. Significant variability was noted in the forms of data presentation of measured EE: it was presented in various units as well as in various statistical forms (average, median), which makes it impossible to compare the presented results or their meta-analysis. In addition, not all studies measured EE as REE, although according to study protocols, including suspension of nutritional support a few hours before testing, suggests that the measured EE corresponds to REE. Comparing individual works is also hampered by incomplete information pertaining to the characteristics of the studied groups with respect to parameters, such as sex, body weight, BMI, and severity of the patient’s condition (assessed on the APACHE II scale), which also affect EE. In displaying parameters describing the studied groups, the formula may be the work of Takemae et al. [52] who presented the detailed characteristics of the studied groups separately for women and separately for men, which may be important in studies of metabolism and EE. The advantage is that in most of the studies analysed, the APACHE II scale was chosen as an indicator of the severity of the patient’s condition, which facilitated the analysis of results.

A total of 12 of the 14 analysed studies provided information on criteria for inclusion and exclusion of participants from the studies. This information is valuable for two reasons: firstly, it allows for even more accurate characteristics of the examined group to be included, and secondly, it assures the correctness of the results obtained; n the case of the analysed works, many of the exclusion criteria were directly related to IC technical limitations, and the lack of exclusion would thus lead to unreliable measurements.

Furthermore, information on the protocol for measuring EE by IC varied from one work to another. High volatility was characterised by, among other factors, the number of hours for which nutrition was suspended, which may be important for the results obtained (for both REE and RQ). It should be emphasised that the studies with the longest period of feeding suspension (up to nine hours–Rusavy et al. [62] and all-night fasting–Saeed et al. [63]) concerned carbohydrate metabolism, which may be the reason for such a long suspension of nutrition. Moreover, not all works contained information about device calibration performance, measurement duration, and achievement of the steady state, which are important for the possible use of test results as a reference point or inclusion in meta-analyses. Oshima et al. [10] have proposed checkpoints in a list for a successful IC that can be easily adapted to develop a protocol for measuring EE by IC in patients.

Another important aspect that appeared in most studies was the day of illness on which the measurement was performed. This information is particularly important for high-dynamic diseases such as sepsis because it has been shown (including in the Uehara et al. [60] study analysed in this review) that EE is characterised by variability depending on the stage of the disease. However, as noted in two studies, some limitations should be taken into account, namely, that patients can be admitted to the ICU at various stages of the disease, after having been initially treated in another ward. This problem can be addressed, e.g., by analysing the patient’s medical history. It is also valuable to know whether IC is performed in patients undergoing mechanical ventilation, both due to a different measurement technique, and the demonstrated impact of liberation from mechanical ventilation on the REE of patients with sepsis [64].

### 5.3. Definition of Sepsis

**Figure 5:**
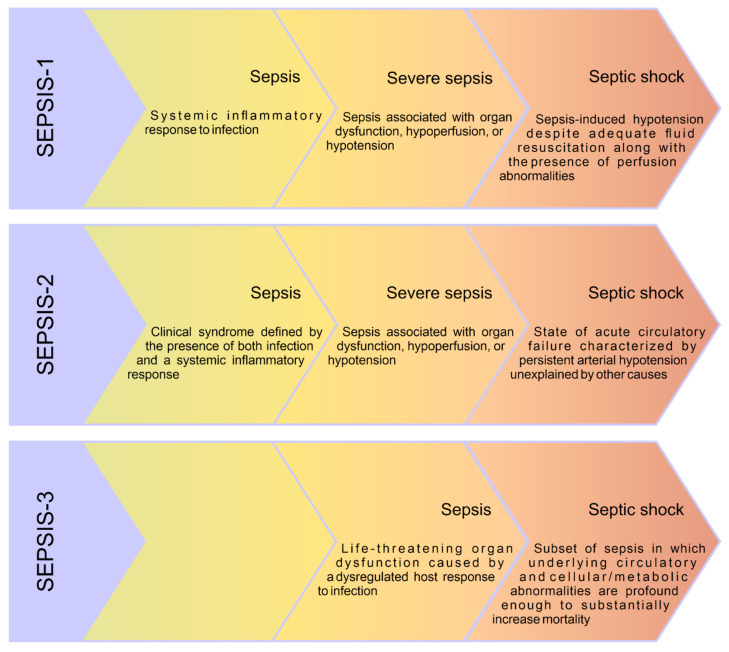
Comparison of old and new sepsis definitions. The first definition of sepsis and classification of clinical conditions associated with it were presented as a result of the American College of Chest Physicians (ACCP) and Society of Critical Care Medicine (SCCM) conference in 1991 (SEPSIS-1) [71]. Due to reservations, mainly related to the non-specificity of the definition of systemic inflammatory response syndrome (SIRS), a conference called SEPSIS-2 was organised in 2001, during which the definitions established during SEPSIS-1 were maintained and an extended list of possible symptoms of systemic inflammation in response to infection was proposed [68]. A further need to update the sepsis nomenclature led to the publication of The Third International Consensus Definitions for Sepsis and Septic Shock (SEPSIS-3) in 2016, including new definitions of sepsis and septic shock [1].

In addition, when analysing the results of published studies, attention should be paid to the definition used for sepsis. As described in the introduction, both the view on pathophysiology and the definitions of sepsis and septic shock have changed over the years. Before comparing the results obtained in different studies, it is necessary to make sure that they actually relate to the same population, e.g., the definition of severe sepsis, appearing in research from a few years ago, corresponds to the current definition of sepsis [1]. The evolution of the definition of sepsis in modern times is presented in Figure 5.

### 5.4. Limitations of the Discribed Studies

In addition to concerns about not knowing the stage of the disease and associated physiological and therapeutic parameters, other study limitations explained by the authors of the studies included that fact that the research was conducted at a single centre and that the size of the examined group was small. Data associated with change in REE [5] were not presented. 

Unification of the protocol for measuring EE by IC, as well as the form and detail of data presentation, would overcome this limitation through a meta-analysis of the results of research conducted across many centres; this can prove to be a milestone in research on energy metabolism in sepsis.

It is worth remembering that the EE measurement was only a tool, and not a goal in the research works discussed. Therefore, further studies with EE as a primary outcome parameter should be performed to shed light on this issue.

### 5.5. Limitations of This Review

This work has some limitations, which include a review of literature within only one database and the use of a search formula covering only titles and abstracts, which may result in the exclusion of some studies. However, this does not appear to significantly change the results obtained; a review within one database allowed for the analysis of many aspects of the methodology of studies using indirect calorimetry in patients with sepsis and to identify potential problems hindering the meta-analysis of these studies.

## 6. Conclusions

Sepsis, as a condition in which severe metabolic disorders occur, requires adequate nutritional support. The current nutritional strategy for these patients is focused on EN optimisation, and IC is considered the gold standard in assessing energy requirements. IC is a valuable method for optimising nutritional care in critically ill patients. There are studies assessing the EE of patients with sepsis by IC, but due to differences in data presentation and study protocols, their collective analysis is difficult. Due to the common study limitations of single centres and small study group sizes across the studies analysed, a meta-analysis of the results could enable evaluation covering many centres and a much larger group of patients. Standardisation of the research protocol and the form and manner in which results are presented, would allow a comprehensive meta-analysis of the data to provide deeper insights into energy metabolism in sepsis. Furthermore, the limitations discussed above should prompt scientists to thoughtfully design clinical trials, and clinicians to interpret results with care.

## Figures and Tables

**Table 1:** Summary of studies in patients with sepsis/septic shock in which indirect calorimetry was used.

Reference	Type of Study	Objective of the Study	Only Septic Patients
Takemae et al. (2020) [52]	Retrospective observational study	Development of new equations to estimate the total EE of Japanese patients with sepsis.	Yes
Menegueti et al. (2019) [53]	Observational cross-sectional study	Assessment of whether REE, respiratory quotient, oxygen consumption, and carbon dioxide production (measured by IC) differ in critically ill patients with sepsis compared to critically ill patients without sepsis.	No
Panitchote et al. (2017) [54]	Prospective observational study	Assessment of the correlation between REE of patients with sepsis/septic shock, measured by IC and estimated using predictive equations.	Yes
Lee et al. (2017) [64]	ND	Identification of the difference in EE and substrate utilisation by patients during and upon liberation from mechanical ventilation.	Yes
Wu et al. (2016) [65]	Prospective observational study	Assessment of the short-term consequence of continuous renal replacement therapy on body composition and pattern of EE.	Yes
Wu et al. (2015) [55]	Prospective observational study	Assessment of the incidence of hypermetabolism, defined as high REE, in severe sepsis ICU patients, and evaluate the suitability of excessive RRE as a risk factor of their clinical outcome.	Yes
Hickmann et al. (2014) [66]	Prospective observational study	Determining the impact of early exercise on energy requirements to adjust caloric intake accordingly in critically ill patients.	No
Auxiliadora-Martins et al. (2008) [43]	Prospective clinical study	Comparison of two different CO monitoring systems based on the thermodilution principle (Thermo-CO) and IC (Fick mixed-CO) in septic patients.	Yes
Basile-Filho et al. (2008) [56]	Prospective clinical study	Comparison of REE obtained by IC and the REE calculated by predictive equations (Brandi and Liggett) using the oxygen consumption obtained by Fick‘s method in septic patients.	Yes
Auxiliadora-Martins et al. (2008) [57]	Prospective clinical study	Evaluation of the ^13^CO_2_ recovery fraction in expired air after continuous intravenous infusion of NaH^13^CO_2_, in critically ill patients with sepsis under mechanical ventilation (calculation of substrate oxidation).	Yes
Gore et al. (2006) [67]	ND	Investigating the haemodynamic and metabolic effects of cardiac selective beta adrenergic blockade in septic patients.	Yes
Dvir et al. (2006) [58]	Prospective observational study	Measuring the daily cumulative energy balance in critically ill patients receiving mechanical ventilation using a bedside computerised information system, and to assess its impact on outcome.	No
Rusavy et al. (2005) [61]	ND	Comparing the effects of 2 blood glucose levels (5 and 10 mmol/L) under hyperinsulinemic conditions, and the effect of glycaemia 5 mmol/L with extremely high insulinaemia on glucose metabolism and EE in septic patients.	Yes *
Natalini et al. (2005) [44]	Open-label, controlled clinical trial	Comparison of the effects of noradrenaline and metaraminol on haemodynamics in septic shock patients.	Yes
Rusavy et al. (2004) [62]	ND	Comparing the effects of two levels of insulinaemia on glucose metabolism and EE in septic patients and volunteers.	Yes *
Marson et al. (2004) [45]	Prospective study	Comparison of oxygen consumption index measured by using IC with a portable metabolic cart and calculated according to Fick‘s principle in critically ill patients.	No
Fernandes et al. (2001) [46]	Interventional, prospective, randomised, controlled study	Evaluation of the haemodynamic and oxygen utilisation effects of haemoglobin infusion on critically ill septic patients.	Yes
Sakka et al. (2001) [47]	Prospective clinical study	Examining the variability of splanchnic blood flow during a 4-h period of unchanged global haemodynamics in patients with sepsis.	Yes
Zauner et al. (2001) [59]	Prospective, clinical cohort study	Evaluation of the energy and substrate metabolism in septic and non-septic critically ill patients in the resting state and during the administration of standardised total parenteral nutrition.	No
Schaffartzik et al. (2000) [48]	Prospective clinical study	Comparison of oxygen consumption obtained from breathing gases by IC with a metabolic monitor integrated with a respirator and oxygen consumption obtained by the Fick principle in patients with sepsis after an increase in oxygen delivery induced by positive inotropic support.	Yes
Broccard et al. (2000) [49]	ND	Evaluation of the tissue oxygenation and haemodynamic effects of NOS inhibition in clinical severe septic shock.	Yes
Sakka et al. (2000) [50]	Prospective clinical study	Comparison of four clinical techniques of measuring cardiac output in critically ill patients: pulmonary artery thermodilution, transpulmonary aortic thermodilution, Fick principle-derived, and continuous pulmonary artery measurements.	Yes
Opdam et al. (2000) [51]	Prospective observational study	Determining whether there is a correlation between lung lactate release and lung oxygen consumption by studying adult intensive care patients, either after cardiopulmonary bypass or with septic shock.	No
Uehara et al. (1999) [60]	Prospective study	Obtaining accurate values for the components of EE in critically ill patients with sepsis or trauma during the first 2 weeks after admission to the ICU.	No
Saeed et al. (1999) [63]	ND	Assessment of the effect of sepsis on total glucose utilisation, oxidation and storage, and the energetic costs of these metabolic processes.	Yes *

* Septic patients and healthy volunteers as a control group. The content of the table contains quoted information from the articles, with possible modifications. The type of study was categorised according to the study authors’ declarations. Abbreviations: ^13^C—labeled carbon; CO—cardiac output; CO_2_—carbon dioxide; EE—energy expenditure; IC—indirect calorimetry; ICU—Intensive care unit; NaH^13^CO_2_—labeled bicarbonate; ND—no data; NOS—nitric oxide synthase; REE—resting energy expenditure; RQ—respiratory quotient.

**Table 2:** Comparison of results and some aspects of the methodology in studies using indirect calorimetry in patients with sepsis or septic shock.

Reference	Diagnosis	Criteria for Sepsis AND Septic Shock	Sample Size	% of Women	Age (years)	Body Mass (kg)	BMI (kg/m^2^)	APACHE II (points)	Mechanical Ventilation (%)	Device	Nutrition during IC	Day of Measurement	EE (kcal/24 h)	EE (kcal/kg/24 h)	RQ
Takemae et al. (2020) [52]	Severe sepsis	SEPSIS-2 [68] SSC 2012 [69]	42	0%	68 ± 14	60 ± 14	22.2 ± 4.7	24.2 ± 5.8	100%	M-COVX^®^ (Datex-Ohmeda, Helsinki, Finland)	≥4 h between changes in the feeding method and IC	1st day of the intubation period	ND	ND	0.78 ± 0.09
			24	100%	60 ± 16	48 ± 16	20.4 ± 5.3	27.6 ± 6.0							0.77 ± 0.9
			19	0%	66 ± 13	62 ± 10	23.0 ± 2.9	26.9 ± 5.7							0.81 ± 0.11
			10	100%	56 ± 15	60 ± 17	25.1 ± 7.1	34.8 ± 8.0							0.76 ± 0.12
Menegueti et al. (2019) [53]	Sepsis/septic shock	SSC 2008 [70]	91	42%	58 (19–89) ^m(r)^	ND	26 (17–45) ^m(r)^	25 (9–47) ^m(r)^	100%	Deltatrac II^®^ (Datex-Ohmeda)	IC before the beginning of nutrition	First 48 h of admission	1430 (540–2420) ^m(r)^	ND	0.82 (0.6–1.24) ^m(r)^
Panitchote et al. (2017) [54]	Severe sepsis/septic shock	ND	16	44%	71.6 ± 5.5	ND	22.0 ± 2.9	26.9 ± 4.0	100%	Engström Carestation^®^ (GE Healthcare, Chicago, IL, USA)	ND	24 h	1488 ± 261	26.7 ± 5.3	ND
												48 h	1459 ± 270		
												72 h	1560 ± 363		
Lee et al. (2017) [64]	Septic shock	ND	37	43%	69 ± 10	59.01 ± 7.63	ND	22 ^m^	100%	CCM Express^®^ (Medical Graphics Corporation, St Paul, MN, USA)	Suspended 4 h before IC	ND	2090 ± 489	ND	ND
Wu et al. (2016) [65]	Sepsis and CRRT requirement	SSC 2012 [69]	27	41%	48.2 ± 22.0	62.8 ± 14.7	22.0 ± 1.4	ND	48.1%	Metabolic cart (Cosmed, Roma, Italy)	Suspended ≥1.5 h before IC	At admission	ND	27.9 ± 5.9	0.81 ± 0.06
												Before CRRT ^a^		29.9 ± 5.6	0.82 ± 0.06
												6 h after CRRT ^a^		26.6 ± 4.3	0.86 ± 0.05
Wu et al. (2015) [55]	Severe sepsis/septic shock	SSC 2012 [69]	62	35%	57.1 ± 19.5	79.1 ± 10.3	21.6 ± 3.1	20.2 ± 4.1	37.5%	Metabolic cart (Med Graphics)	Suspended ≥1.5 h before IC	1st, 2nd, 3rd, 4th, 5th day	ND	ND	ND
Basile-Filho et al. (2008) [56]	Septic shock	SEPSIS-1 [71]	15	27%	41.3 ± 18.9	68.5 ± 9.2	ND	22.6 ± 7.2	100%	Deltatrac II^®^ (Datex–Ohmeda)	ND	3rd–5th day	1669 ± 271	ND	0.82 ± 0.11
Auxiliadora-Martins et al. (2008) [57]	Sepsis/septic shock	SEPSIS-1 [71]	10	60%	55.1 ± 19	ND	ND	25.9 ± 7.4	100%	Deltatrac II^®^ (Datex-Ohmeda)	ND	2nd–5th day	1587 ± 430 ^b^	ND	0.79 ± 0.10
Gore et al. (2006) [67]	Sepsis	ND	6	ND	41 ± 7	81 ± 18	ND	17 ± 2	100%	Delta Trac^®^ (Sensormedics, Yorba Linda, CA, USA)	EN 40 cal/h during IC	ND	1414 ± 134	ND	0.99 ± 0.06
Rusavy et al. (2005) [61]	Sepsis	ND	10	ND	ND	ND	ND	18.4 ± 2.12	100%	Deltatrac II^®^ (Datex, Instrumentarium, Helsinki, Finland)	ND	ND	2179± 354	ND	ND
Rusavy et al. (2004) [62]	Sepsis	ND	20	ND	65 (52–68) ^m(IQR)^	ND	26 (24.6–27.8) ^m(IQR)^	20.2 (18.3–22.4) ^m(IQR)^	100%	Deltatrac II® (Datex-Ohmeda)	Suspended 9 h before IC	3rd–7th day	2116 (1880–2455) ^m(IQR)^	ND	0.79 (0.77–0.85) ^m(IQR)^
Zauner et al. (2001) [59]	Severe sepsis/septic shock	SEPSIS-1 [71]	14	43%	57.5 ± 12.92	71.4 ± 12.7	24.1 ± 4.2	ND ^c^	ND	MMC 2900^®^ (SensorMedics)	TPN was started after the first IC	At admission	ND ^d^	ND	0.77 ± 0.05
												2nd day			0.84 ± 0.05
												7th day			0.86 ± 0.05
Uehara et al. (1999) [60]	Severe sepsis	SEPSIS-1 [71]	12	33%	67 (25–84) ^m(r)^	Day 0: 78.4 ± 3.8 Day 5: 74.2 ± 3.3 Day 10: 70.2 ± 3.4 ^mean±SEM^	ND	23 (15–34) ^m(r)^	100%	Deltatrac MBM-100^®^ (Datex/Instrumentarium)	ND	2nd 3rd 4th 5t 6th 7th 8th 9th 10th 11th 12th 23rd day	1859 ± 140 1840 ± 119 1864 ± 139 1988 ± 121 2047 ± 141 2091 ± 140 2022 ± 150 2061 ± 138 2036 ± 147 1947 ± 126 2013 ± 140 1770 ± 116 ^mean±SEM^	ND	ND
Saeed et al. (1999) [63]	Sepsis	SEPSIS-1 [71]	24	42%	52.2 ± 15.6	77.2 ± 11.7	ND	ND	ND	Deltratrac^®^ (Datex)	PN overnight fast before IC	ND	ND ^e^	ND	ND

^a^ Length of ICU stay before CRRT, days (mean ± SD) 6.7±4.8. ^b^ 1587 ± 430 kcal/min according to the authors, probably a mistake in terms of time unit. ^c^ The APACHE III score was used (70.2 ± 11.1). ^d^ The results are given as kJ · min^−1^ m^−2^ (Day 0—2.65 ± 0.5; Day 2—2.69 ± 0.5; Day 7—2.55 ± 0.7). ^e^ REE was expressed relative to FFM (kcal per kg FFM per min). ^m(r)^ Median (range). ^m(IQR)^ Median (IQR). The values in the table are given as mean ± SD, unless otherwise stated. Abbreviations: APACHE II—Acute Physiology and Chronic Health Evaluation II; BMI—body mass index; CRRT—continuous renal replacement therapy; EN—enteral nutrition; FFM—fat free mass; IC—indirect calorimetry; IQR—interquartile range; ND—no data; PN—parenteral nutrition; REE—resting energy expenditure; RQ—respiratory quotient; SD—standard deviation; SEM—standard error of the mean; TPN—total parenteral nutrition.

**Table 3:** Summary of study exclusion criteria.

	Reference	Takemae et al. (2020) [52]	Menegueti et al. (2019) [53]	Panitchote et al. (2017) [54]	Lee et al. (2017) [64]	Wu et al. (2016) ^b^ [65]	Wu et al. (2015) [55]	Basile-Filho et al. (2008) [56]	Auxiliadora-Martins et al. (2008) [57]	Gore et al. (2006) ^c^ [67]	Rusavy et al. (2005) ^d^ [61]	Rusavy et al. (2004) ^d^ [62]	Zauner et al. (2001) [59]	Uehara et al. (1999) ^e^ [60]	Saeed et al. (1999) ^d^ [63]
Exclusion Criterion ^a^															
Age (years)		<18		<18	<18	<18	<18	<15 >80	<15 >85						
Chest tube/drain		+	+	+											
Bronchopleural fistula				+											
PEEP (cm H_2_O)		>12	>14	>12			>12								
FiO_2_		≥0.6	>0.6	>0.6		>0.6	> 0.6	>0.6	>0.6			>0.7	>0.55		
MAP (mm Hg)								<50	<50		<70	<75			
Diuresis (ml/h)								<50	<50						
Cardiac index											<3	<3			
Respiratory rate (breath/min)		>35													
Lactate (mmol/L)											↑ trend	↑ trend	>5		
Changes in buffer base in 12 h											>10%	>10%			
Haemodialysis		+		+							+	+			
CRRT		+													
ECMO		+													
Brain death			+						+						
Pregnancy						+	+								
Endocrine/metabolic disorders							+						+		+
Triacylglycerol (mmol/L)													>5.1		
Oliguric renal insufficiency								+							
Haemodynamic shock													+		
Major pulmonary complications					+										
Malignant disease															+
Significant postoperative bleeding					+										
Isolation protocol					+										
Comfort care directives					+										
Expected ICU stay (days)							<5								
Corticosteroid treatment											+				+
Catecholamine treatment											+				
β-adrenoceptor antagonist treatment															+
Thyroid hormones treatment															+
Clinical conditions resulting in false data of body composition parameters						+									
Refusal to participate									+						

^a^ Some exclusion criteria are based on inclusion criteria. ^b^ Body composition was also assessed. ^c^ The authors do not provide criteria for inclusion and exclusion from the study; a brief description of patients is available: *All subjects had a MAP > 70 mm Hg without inotropic support. Urine output > 0.5 cc/kg/hour on all subjects at the time of study. No subject was hypoxic (O_2_ saturation ≤ 94%) or severely acidotic (pH ≤ 7.32)*. ^d^ Glucose metabolism was also assessed. ^e^ The authors do not provide inclusion and exclusion criteria except *criteria for entry into this study, for patients with sepsis, were those of the ACCP/SCCM Consensus Conference*. +, the criterion was used in the study; ↑, increasing. Abbreviations: CRRT—continuous renal replacement therapy; ECMO—extracorporeal membrane oxygenation; FiO_2_—fraction of inspired oxygen; ICU—Intensive care unit; MAP—mean arterial pressure; PEEP—positive end-expiratory pressure.

**Table 4:** Limitations of the analysed studies.

Reference	Limitations
Takemae et al. (2020) [52]	No specific protocol to control nutrition during patient intubation; A small number of REE data were acquired per day.
Menegueti et al. (2019) [53]	The REE was measured only at admission to the ICU; The study was conducted in a single centre
Panitchote et al. (2017) [54]	Difficulties in obtaining steady state; The small sample size; The IC was measured only 6 h per day and did not occur randomly during the day; Activities were not recorded during the measurements.
Lee et al. (2017) [64]	Heterogeneous nature of the cohort; Patients whose disease progression warrants admission to the ICU can be in their late and more severe stages; Variability in sedation management and body mass.
Wu et al. (2016) [65]	A short-term self-control study in surgical ICU–mortality outcomes of enrolled patients were not followed; A small-size study at a single department; Plasma cytokine concentration and ultrafiltration were not tested due to operational difficulties.
Wu et al. (2015) [55]	The effect of medical procedures on the REE determination has not been evaluated in each individual patient included; The IC measurement was performed around noon every day; A single centre, small sample study; Some patients entered the ICU directly without prior hospitalisation, while others were admitted from the ward or postoperatively–the included patients were at various stages in the course of their disease.
Basile-Filho et al. (2008) [56]	ND
Auxiliadora-Martins et al. (2008) [57]	ND
Gore et al. (2006) [67]	ND
Rusavy et al. (2005) [61]	ND
Rusavy et al. (2004) [62]	The volunteers were younger, and had lower fasting glycaemia and EE–increased age decreases insulin sensitivity; Calculation of carbohydrate and fat utilisation on the basis of nonprotein RQ can lead to errors if the rates of gluconeogenesis and ketogenesis are changing.
Zauner et al. (2001) [59]	ND
Uehara et al. (1999) [60]	ND
Saeed et al. (1999) [63]	ND

The table presents the limitations of the analysed studies provided by the authors. The content of the table contains direct information quoted from articles, with possible modifications. Abbreviations: EE—energy expenditure; IC—indirect calorimetry; ICU—Intensive care unit; ND—no data; REE—resting energy expenditure; RQ—respiratory quotient.

## Data Availability

Not applicable.
